# Renal Trapping in Accidental Metformin Intoxication

**DOI:** 10.1016/j.ekir.2020.06.009

**Published:** 2020-06-18

**Authors:** Rene A. Posma, A. Mireille A. Wessels, Willem Dieperink, Jan Roggeveld, Henri G.D. Leuvenink, Iwan C.C. van der Horst, Wilfred F.A. den Dunnen, Maarten W. Nijsten, Daan J. Touw

**Affiliations:** 1 Department of Critical Care, University of Groningen, University Medical Center Groningen, Groningen, The Netherlands; 2 Department of Clinical Pharmacy and Pharmacology, University of Groningen, University Medical Center Groningen, Groningen, The Netherlands; 3 Department of Surgery, University of Groningen, University Medical Center Groningen, Groningen, The Netherlands; 4 Department of Intensive Care, Maastricht University Medical Center+, Maastricht University, Maastricht, The Netherlands; 5 Department of Pathology, University of Groningen, University Medical Center Groningen, Groningen, The Netherlands

Metformin is widely used as an antihyperglycemic drug to treat patients with type 2 diabetes. Because metformin is renally excreted and not metabolized, it can accumulate in patients with renal insufficiency and cause lactic acidosis, known as metformin-associated lactic acidosis (MALA).^1^^,^^2^ The reported incidence of MALA ranges from 3 to 10 per 100,000 patient-years and is associated with a high mortality rate. However, the full clinical context or metformin blood concentration is often not reported, making it challenging to distinguish metformin-associated from metformin-induced lactic acidosis (MILA), respectively.^1^

Normally, metformin shows 2-compartment pharmacokinetics with a terminal half-life of 20 hours, suggesting the existence of a deeper compartment.^2^ After oral administration to mice, accumulation of metformin was observed in the gut, kidneys, and liver.^3^ After 6 to 8 weeks of metformin therapy given to drug-naive patients with type 2 diabetes, the metformin level in the jejunum was about 30 to 300 times higher than plasma concentrations.^4^ During MALA, higher metformin concentrations have been measured in erythrocytes compared with plasma, and the drug remains detectable in plasma up to 3 weeks thereafter, suggesting the sustained release of metformin from deeper compartments to the extracellular fluid.^5^ Thirty-seven hours after a patient was admitted because of intentional metformin intoxication, approximately 2 and 20 times the plasma concentration of metformin was found in liver and kidney tissue, respectively.^6^ However, a similar metformin level in liver tissue compared to the plasma concentration after intentional metformin intoxication was also reported.^7^ Metformin tissue levels of patients presenting with accidental metformin intoxication are unknown. Here, we report the clinical course and autopsy of a patient admitted to the intensive care unit (ICU) with accidental metformin intoxication.

Four days after the onset of sharp abdominal pain, nausea and vomiting, and, at times bloody, diarrhea, the patient presented at the emergency department of another hospital. Upon initial assessment, the patient was anuric, and laboratory tests showed acute renal failure (creatinine 903 μmol/l) and severe metabolic acidosis (pH 7.04, bicarbonate 4 mmol/l, and lactate 11.5 mmol/l). Sodium bicarbonate 1.4% infusion was initiated, and 4 hours after admission to the referring hospital, the patient was transferred to our hospital in order to start acute dialysis. Subsequently, the patient was admitted to our ICU with acute renal failure and severe lactic acidosis due to suspected metformin intoxication and septic shock (Figure 1a−c). At admission to the ICU, metformin plasma concentration was 24.6 mg/l, which is 5 times the metformin concentration often considered being the toxic threshold (5 mg/l).^2^^,^^8^ The plasma concentration was twice the whole blood concentration (Figure 1c). After initiating hemodialysis using a low-flux dialyzer (Polyflux 17L, Baxter, Utrecht, The Netherlands) with a blood flow ranging from 200 to 300 ml/min and a dialysate flow of 500 ml/min, respectively, metformin plasma concentration declined more rapidly than the metformin concentration in whole blood, consistent with previous reports.^4^ After cessation of hemodialysis, continuous venovenous hemofiltration was initiated to prevent potential drug rebound.^9^^,^S1 As metformin levels were not readily available, increased metabolic derangement during this period (Figure 1a and b) gave rise to clinical suspicion for a rebound effect. Subsequently, hemodialysis was administered for another 2 hours with similar dialysis settings, followed by 22 hours of continuous renal replacement therapy. Blood and stool cultures were both positive for *Salmonella enterica.* The patient was treated with norepinephrine at a maximum dose of 0.44 μg/kg per minute as vasopressor while remaining anuric throughout ICU admission, and ultimately died of multiorgan failure 39 hours after ICU admission.

**Figure 1:**
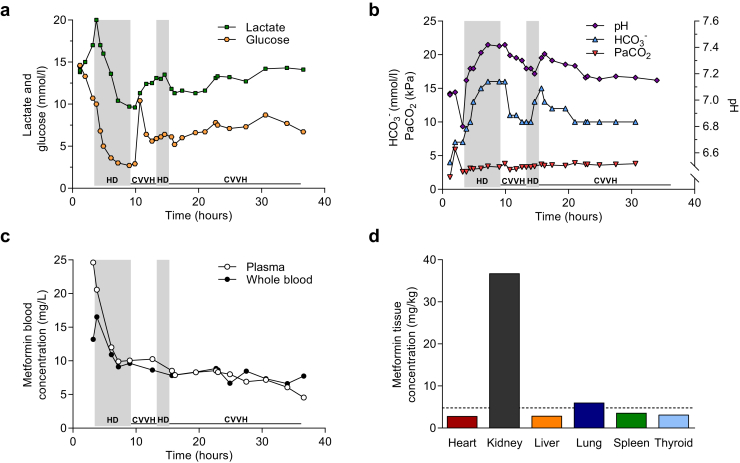
(a) Lactate and glucose levels, and (b) pH, bicarbonate (HCO_3_^–^), and arterial partial pressure of CO_2_ (PaCO_2_) measured by blood gas analysis (ABL90 FLEX, Radiometer, Bronshoj, Denmark) during intensive care unit (ICU) admission (to convert PCO_2_ to mm Hg, multiply values in kPa by 7.5). The shaded gray area denotes the periods of intermittent hemodialysis (HD); continuous venovenous hemofiltration (CVVH) was performed during the remaining time. (c) Metformin concentration in plasma and whole blood during ICU admission (to convert metformin concentration to μmol/l, multiply values in mg/l by 7.74). (d) Metformin level in homogenized tissue samples of several organs that were obtained after an autopsy was performed. The dashed line represents the last plasma metformin concentration; the black bars represent a single value per organ. The patient died within 3 hours after the last blood sample was collected.

In hindsight, we did not observe a rebound in drug levels after cessation of the first hemodialysis session, although lactate levels did increase considerably during this period (Figure 1a). The metformin concentration did not increase after dialysis was stopped, refuting the initial hypothesis of drug rebound. Therefore, the increasing lactate levels probably resulted from pharmacodynamic effects of metformin or metformin-independent causes for increased production or reduced clearance of lactate. As massively elevated aminotransferases (aspartate transaminase >19,000 U/l and alanine transaminase >4,000 U/l, respectively) were also accompanied by marked hypoglycemia (Figure 1a) and emerging coagulopathy, reduced lactate clearance due to acute liver failure seems to be a more likely explanation for the metabolic deterioration during this period.

During the autopsy, hepatic steatosis and signs of ischemic necrosis in zone 3 of the liver as well as renal tubular necrosis were observed. There was no evidence for gastrointestinal perforation or intestinal ischemia. The metformin level in renal tissue was 8-fold higher (36.8 mg/kg, Figure 1c) than the last plasma concentration. The metformin level in other organs, including the liver, approximated the plasma concentration, indicating that metformin equilibrated with the circulating concentration for these organs.

Because the metformin concentration plateaued between hemodialysis sessions, metformin clearance by renal replacement therapy has to be within the same range as the sum of potential redistribution from a deeper compartment and absorption from the gut, considering negligible renal clearance under anuria. As it has been previously reported that metformin clearance by continuous renal replacement therapy ranges from 9 to 71 to ml/min,^9^^,^S1 the corresponding influx should be at a similar rate. Based on the findings of the current study, however, we cannot distinguish to what extent redistribution or absorption contributed to the influx of metformin.

Metformin transport within the kidney indirectly requires energy. Metformin is transported into proximal tubule cells by organic cation transporter 2 and is primarily excreted into the urine by the proton-antiporter multidrug and extrusion transporter (MATE) 2.^2^ MATE-1 dysfunction in mice, a species in which MATE-2 is not expressed, caused accumulation of metformin in liver and kidney tissue and led to lactic acidosis.S2 Likewise, inhibition of MATE-1 by atenolol increased metformin levels in rat kidneys.S3

As the metformin level was elevated in kidney tissue, the efflux of metformin from the proximal tubules into urine or back into the circulation could be hampered in our patient. Renal secretory and subsequent drainage mechanisms were then thus overwhelmed and were neither able to sufficiently eliminate metformin into urine nor able to redistribute metformin to other compartments despite the presence of a concentration gradient after removal of metformin from the circulation by dialysis. Being a mild but specific mitochondrial inhibitor,S4−S7 it is unknown whether metformin in itself plays a role in this process.

Our study has some limitations that have to be pointed out. As it concerns a case report presenting a finding that has not been reported previously, it is unknown whether our results are generalizable to patients using metformin as regular treatment and, specifically, to patients with MALA or MILA. Moreover, we measured tissue metformin levels at only one timepoint, and therefore we cannot report longitudinal data of metformin distribution for each organ. Based on the respective causal roles of underlying systemic diseases and of metformin regarding the development of lactic acidosis, different definitions have been proposed to describe this phenomenon.^1^ Depending on the presence of other pathophysiological conditions and the blood metformin concentration, lactic acidosis is regarded as being not related to, associated with, or induced by metformin therapy.^1^ As this patient previously had a normal renal function and was admitted with an advanced stage of sepsis with renal and liver failure, we cannot ascertain conclusively the contribution of metformin toxicity to the degree of lactic acidosis or cause of death.

In conclusion, our study shows that, in contrast with the metformin level in other tissues and plasma, metformin markedly accumulated in renal tissue. We hypothesize that metformin did not redistribute from the kidneys into the extracellular fluid, while its concentration in blood declined due to prolonged renal replacement therapy. Because tissue of other organs had metformin levels comparable with the last plasma concentration, we believe that metformin equilibrated with the circulating concentration for these organs, whereas this does not happen for the kidney, which thus can play a role in the development of accidental metformin intoxication.

## Disclosure

All the authors declared no competing interests.
