# Supplementary material for: Renal Trapping in Accidental Metformin Intoxication
Source: Kidney Int Rep. 2020 Jun 18;5(9):1525–8. doi: 10.1016/j.ekir.2020.06.009 (PMC7486141; doi:10.1016/j.ekir.2020.06.009)
Supplement: Supplementary File (PDF) [file mmc1.pdf]

Supplemental Material

Renal Trapping in Accidental Metformin Intoxication

Rene A. Posma, A. Mireille A. Wessels, Willem Dieperink, Jan Roggeveld, Henri G.D. Leuvenink, Iwan C.C. van der Horst, Wilfred F.A. den Dunnen, Maarten W. Nijsten, Daan J. Touw

Supplemental methods

We report the clinical course and autopsy of a patient admitted to the intensive care unit (ICU) with an accidental metformin intoxication. Written informed consent was provided for the collection of residual material, including blood. After the patient was deceased, relatives consented to perform autopsy. Ethical approval was given by the institutional review board (METc 2014-552), and the study was carried out according to the principles of the Declaration of Helsinki as revised in 2008.

After blood gas analysis was performed using an ABL90 FLEX as part of routine clinical care, anticoagulated blood was collected from safePICO syringes (Radiometer, Brønshøj, Denmark). Whole blood and plasma, the supernatant of whole blood centrifuged at 1000g for 12 minutes, were stored at -80°C for further analysis. After performing autopsy according to local standard procedures, samples of heart, kidney, liver, lung, and spleen tissue were obtained and stored at -80°C. Subsequently, tissue samples were homogenised to a fine powder at liquid nitrogen temperature using mortar and pestle.

For all specimens, a sample volume of 10 µl was mixed with 750 µl of a mixture of methanol and acetonitrile (4:21, vol/vol) and $^{2}H_{6}$-metformin 0.1 mg/L (Ritmeester, Nieuwegein, the Netherlands). To determine the metformin concentration with liquid chromatography-tandem mass spectrometry, the mixed samples were centrifuged at 10,000g for 5 minutes, and 5 µl of the supernatant was injected into a triple-stage quadrupole Quantum Access Max mass spectrometer coupled to a Vanquish ultra-performance liquid chromatography pump, autosampler and column oven (Thermo Scientific, San Jose, CA, USA). The lower limit of quantitation was 0.02 mg/L. A tissue density of 1.05 g/cm^3 was assumed for all samples.

Supplemental references

S1. Calello DP, Liu KD, Wiegand TJ, et al. Extracorporeal Treatment for Metformin Poisoning: Systematic Review and Recommendations From the Extracorporeal Treatments in Poisoning Workgroup. *Crit Care Med.* 2015;43:1716-30.

S2. Toyama K, Yonezawa A, Masuda S, et al. Loss of multidrug and toxin extrusion 1 (MATE1) is associated with metformin-induced lactic acidosis. *Br J Pharmacol.* 2012;166:1183-119.

S3. Ma YR, Huang J, Shao YY, et al. Inhibitory effect of atenolol on urinary excretion of metformin via down-regulating multidrug and toxin extrusion protein 1 (rMate1) expression in the kidney of rats. *Eur J Pharm Sci.* 2015;68:18-26

S4. Owen MR, Doran E, Halestrap AP. Evidence that metformin exerts its anti-diabetic effects through inhibition of complex 1 of the mitochondrial respiratory chain. *Biochem J.* 2000;348 Pt 3:607-614

S5. El-Mir MY, Nogueira V, Fontaine E, et al. Dimethylbiguanide inhibits cell respiration via an indirect effect targeted on the respiratory chain complex I. *J Biol Chem.* 2000;275:223-228

S6. Protti A, Fortunato F, Monti M, et al. Metformin overdose, but not lactic acidosis per se, inhibits oxygen consumption in pigs. *Crit Care.* 2012;16:R75

S7. Protti A, Lecchi A, Fortunato F, et al. Metformin overdose causes platelet mitochondrial dysfunction in humans. *Crit Care.* 2012;16:R180

CARE checklis

Topic	Item	Checklist item description	Reported on page
**Title**	**1**	The words “case report” (or “case study”) should be in the title along with phenomenon of greatest interest (e.g., symptom, diagnosis, test, intervention)	1, case report is not specifically mentioned
**Key Words**	**2**	The key elements of this case in 2 to 5 key words, including “case report”	1
**Abstract**	**3a**	Introduction—What is unique about this case? What does it add to the medical literature?	Not applicable
	**3b**	The main symptoms of the patient and the important clinical findings	Not applicable
	**3c**	The main diagnoses, therapeutics interventions, and outcomes	Not applicable
	**3d**	Conclusion—What are the main “take-away” lessons from this case?	Not applicable
**Introduction**	**4**	Brief background summary of this case referencing the relevant medical literature	2
**Patient Information**	**5a**	De-identified patient specific information	2-4
	**5b**	Primary concerns and symptoms of the patient	2,3
	**5c**	Medical, family, and psychosocial history including co-morbidities, and relevant genetic information	5
	**5d**	Relevant past interventions and their outcomes	2-4
**Clinical Findings**	**6**	Describe the relevant physical examination (PE) findings and important clinical findings	PE: not mentioned, clinical findings: 2
**Timeline**	**7**	Depict important milestones related to your diagnoses and interventions (table or figure)	2-3, Supplemental Methods, Fig 1
**Diagnostic Assessment**	**8a**	Diagnostic methods (such as PE, laboratory testing, imaging, questionnaires)	2-3, Supplemental Methods, Fig 1
	**8b**	Diagnostic challenges (such as financial, language, or cultural)	Not mentioned
	**8c**	Diagnostic reasoning including other diagnoses considered	3
	**8d**	Prognostic characteristics (such as staging in oncology) where applicable	Not applicable
**Therapeutic Intervention**	**9a**	Types of intervention (such as pharmacologic, surgical, preventive, self-care)	2-3, Fig 1
	**9b**	Administration of intervention (such as dosage, strength, duration)	2-3, Fig 1
	**9c**	Changes in intervention (with rationale)	Not mentioned
**Follow-up and Outcomes**	**10a**	Clinician-assessed and patient-assessed outcomes (if available)	3,4
	**10b**	Important follow-up and other test results	2-4, Fig 1
	**10c**	Intervention adherence and tolerability (How was this assessed?)	Not applicable
	**10d**	Adverse and unanticipated events	2-4
**Discussion**	**11a**	A scientific discussion of the strengths and limitations associated with this case report	4,5
	**11b**	Discussion of the relevant medical literature with references	4,5
	**11c**	The scientific rationale for any conclusions (including assessment of possible causes)	4
	**11d**	The main “take-away” lessons of this case report in one paragraph conclusion	5
**Patient Perspective**	**12**	Did the patient share his or her perspective or experience? (Include when appropriate)	Not applicable
**Informed Consent**	**13**	Did the patient give informed consent? Please provide if requested	**Yes (including relatives)**
